# Polyphenols in bee products and prevention of cell senescence

**DOI:** 10.37796/2211-8039.1458

**Published:** 2024-09-01

**Authors:** Siti Nuriah M. Noor, Marahaini Musa, Ahmad Azlina, Siew H. Gan, Kannan P. Thirumulu

**Affiliations:** a School of Dental Sciences, Universiti Sains Malaysia, 16150 Kubang Kerian, Kelantan, Malaysia; b Human Genome Centre, School of Medical Sciences, Universiti Sains Malaysia, 16150 Kubang Kerian, Kelantan, Malaysia; c School of Pharmacy, Monash University Malaysia, Jalan Lagoon Selatan, Bandar Sunway, 47500 Selangor Darul Ehsan, Malaysia

**Keywords:** Ageing, Cell senescence, Bee products, Polyphenols, Antioxidant

## Abstract

Sustaining the continuity of cells and their homeostasis throughout the lifespan is compulsory for the survival of an organism. Cellular senescence is one of mechanisms involved in cell homeostasis and survival, and plays both important and detrimental roles in the maintenance of malfunctioned and normal cells. However, when exposed to various insults (genetic, metabolic and environmental), the cells undergo oxidative stress which may induce premature senescence, or so-called stress-induced premature senescence. Many age-related diseases are associated with premature senescence. Hence, there is growing interest in the intake of natural sources such as dietary food, which has protective functions on human health and diseases as well as on premature senescence. There are many natural food sources which have beneficial effects on delaying cell senescence, of which bee products are one of them. Bee products (honey, propolis, royal jelly, bee pollen, bee bread, venom and wax) are rich in polyphenols, a compound that exerts powerful antioxidant actions against oxidative stress and is able to delay premature senescence that is linked to ageing. This review describes the factors triggering senescence, the biomarkers involved and the prevention of senescence by the polyphenols present in bee products. Thus, it is hoped that this will provide new insights into the clinical management of age-related diseases.

## 1. Introduction

Every multicellular organism, such as human, is composed of colonies of cells that are reproduced independently and organised hierarchically, starting with genes to form genomes, and producing eukaryotic cells. Then, millions of cells form the organism [1].

The survival of multicellular organism is a very effective way of living, as it is divided into different cell types with specific functions [2]. In mammals such as human, there are two ways of survival mechanisms; first, specialised cells recognize the malfunctioned cell and destroy it and the second way is based on capability of cells to recognise its own dysfunction and cause its own death [3]. Cellular senescence is an example of mechanism combining both ways in elimination of the cells’ malfunction in mammals [2]. Cellular senescence plays a beneficial and detrimental role on the organism especially in the ageing process [4]. In this review, we describe the cellular senescence including the factors that trigger senescence, biomarkers of cell senescence and the prevention of senescence using polyphenols of bee products.

## 2. What is cell senescence?

Senescence is a type of cellular response characterized by a stable growth arrest and other phenotypic alterations such as profound chromatin and secretome changes, and tumour-suppressor activation [5]. This mechanism was formerly discovered by Leonard Hayflick and Moorhead in 1961 whereby the normal human fibroblasts showed a limited proliferative capacity in culture, recognized as ‘cellular senescence’ and this mechanism might be an underlying cause of ageing [6]. The mechanism of cellular senescence happens when the cells recognise its own dysfunction and induces a stable cell cycle arrest through the activation of cell cycle inhibitors such as p16^INK4A^ and p21^CIP1^. Then, the cells release signals to the immune system in order to recognise and destroy the damaged senescent cells [7]. A permanent growth arrest during senescence helps in prevention of damaged cells or transformed cells from excreting their genomes that might affect other cells [5].

**Fig. 1:**
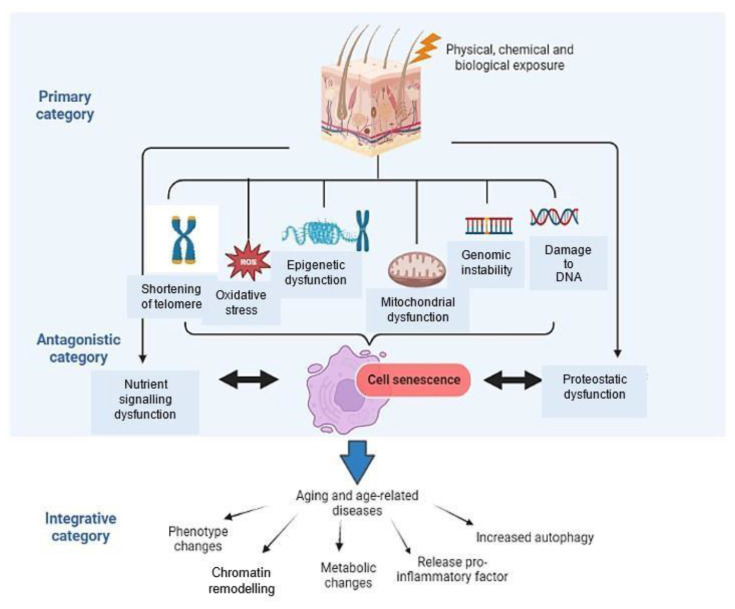
Three categories of ageing hallmarks with senescence as the central hallmark.

Senescence occurs in aged and damaged cells as a response to the damage occurring in damaged cells and in normal cells [8]. Absence of this mechanism in damaged cells plus with aged immunity system that inefficiently removes senescent cells from tissues and insufficient release of senescence associated secretory phenotype (SASP) by the senescent cells contribute to the accumulation of the senescent cells [2]. Overaccumulation of senescent cells promote biological ageing and age-related diseases such as atherosclerosis, diabetes, lung disease, and many others, leading to overall ageing of the organisms [5,9]. Due to this reason, senescence is considered as an ageing hallmark [10]. Senescent cells existing in tissues must be removed in order to delay ageing and extend health span [11]. Additionally, cellular senescence falls under antagonistic category of ageing hallmarks as shown in Fig. 1. There are three categories of ageing hallmarks depicted [5,10] which are 1. Primary - the causes of age related damages such as damage to the telomeres, damage to DNA, and epigenetic and mitochondrial dysfunction, 2. Antagonistic - the responses to the damages such as cell senescence and 3. Integrative - the consequences of the responses and culprits of the ageing phenotype including phenotype changes, chromatin remodelling, metabolic changes and others.

Moreover, senescence also helps in inhibiting tumorigenesis and limiting tissue damage [9]. Besides regulating the damaged cell, senescence is also responsible for the development of normal cells in mammalian embryo and adult tissues [12]. Senescence is responsible for proper tissue remodelling, maintaining tissue homeostasis, removal of unwanted cells during tissue development and regulating proper wound healing by limiting the development of fibrotic tissue [12], while in mature tissues, cellular senescence is mainly triggered by response to injury, thereby inhibiting potentially dysfunctional cells [13].

### 2.1. Factors triggering senescence

Senescence can be initiated by numerous stresses such as telomere shortening, oncogene activation and accumulation of reactive oxygen species (ROS) [8,14]. These stresses were reported to give rise to different types of senescence such as telomere dependent replicative senescence, programmed senescence or non-telomeric stress induced premature senescence including oncogene induced senescence (OIS), unresolved DNA damage induced senescence, epigenetically induced senescence and mitochondrial dysfunction associated senescence [15].

Among those stressors, telomere shortening appears as a top biomarker for ageing [16]. Telomere is the end of linear human chromosomes that consists of tandem repeats of deoxyribonucleic acid (DNA) sequences of TTAGGG which is protected by complex protein, shelterin or telosome and is elongated by telomerase. Telomerase is a reverse transcriptase enzyme that maintains the telomere homeostasis by lengthening the telomeres via adding its RNA template, 5′-TTAGGG-3′ onto newly synthesized DNA at terminal 3′ ends [17]. Telomere helps the chromosomes from fusion with other chromosomes and hence prevents chromosomal instability [18]. With every DNA replication, 50–200 of telomere base pairs (bp) are lost in a human cell due to end replication problem leading to cellular senescence via activation of DNA damage response (DDR) which causes an increase of cellular level of cell cycle inhibitory proteins [19].

Moreover, over expression of oncogenes and inhibition of tumour suppressor genes have induced OIS. Oncogenes are mutated normal genes that are present in the human genome, called proto-oncogenes. This proto-oncogene potentially promotes cancer development, whereas tumour suppressor genes code for proteins that regulate the pathways of prevention for cancer development [2]. Rat sarcoma virus (RAS), protooncogene B-Raf, protein kinase B, E2F Transcription Factor 1 (E2F1) and cyclin-E are examples of oncogenes that are over-expressed in OIS. Additionally, ROS were also suggested to be a factor of cell senescence. Superoxide ion (O2•−), hydroxyl radical (•OH), and hydrogen peroxide (H_2_O_2_) are examples of ROS [2]. ROS are a group of molecules that are formed from mitochondrial oxidation and exogeneous factors such as ultraviolet (UV) radiation and chemicals from tobacco causing damage cellular DNA. Damaged DNA signals a DDR and activates cell cycle inhibitors causing senescence in cells [20].

### 2.2. Biomarkers of cell senescence

It was previously reported that gradual accumulation of senescent cells contributes to ageing. So, it is crucial to identify selective biomarkers to detect the presence of senescent cells in living tissues. However, the biomarkers expressed by the senescent cells might vary with the type of cell, stimulation and stimulation duration [21]. To date, there is no universal marker discovered to detect cell senescence [22]. There are many biomarkers of senescent cells such as increased activity of senescence-associated βgalactosidase (SAβ-gal), increased level of p16^INK4A^, p53, p21^CIP1^, levels of DNA damage including phosphorylated form of histone protein, γ-H2AX, formation of senescence associated heterochromatin foci (SAHF) and secretion of SASP [23]. These biomarkers can also be used as markers in detecting the senescent cells.

Among those biomarkers, activity of SAβ-gal [24] and level of p16^INK4A^ [25,26] are the most widely used biomarkers for evaluating cell senescence. SAβ-gal refers to the cell damage marker and p16^INK4A^ is an inducer and indicative for permanent cell cycle arrest [26]. In addition, senescent cells can also expose their morphological and structural changes, including an enlarged, flattened, multinucleated morphology with enlarged vacuoles [27] altered composition of the plasma membrane and a remarkable nuclear enlargement [28].

### 2.3. Prevention of senescence

Cellular senescence being the main reason that may lead to chronic diseases and age-related maladies, development of interventions that target the senescent cells is vital for therapeutic potential to reduce ageing phenotypes, treat age-related disorders, to improve human lifespan and also to have a healthy lifespan [13,29]. Additionally, many evidences suggest that targeting cellular senescence can significantly improve human health and extend health span which directly increase longevity of life [11]. A report by Campisi et al. [30] mentions that most of the ageing interventions are classified into two categories 1. Involvement of processes aimed in reversing cellular ageing by modulating metabolic pathways or the epigenome and 2. The use of senolytics, a type of drug to eliminate senescent cells by inducing apoptosis.

There are various approaches in delaying senescence using senescent cell models namely vascular endothelial cells (VEC), fibroblasts [31], muscle cells and nerve cells [13]. The approaches include modulating the main senescent pathways such as p16 and p53-p21, the upregulation of Sirtuin 1 (SIRT1), endothelial nitric oxide synthase (eNOS) phosphorylation, superoxide dismutase (SOD), glutathione peroxidase (GSHPx), and transcription factor E2F-1 and the downregulation of microRNA 34a (miR-34a), nuclear factor kappa-light-chain-enhancer of activated B cells (NF-κB), malondialdehyde (MDA) content, and caveolin-1 [13]. Glucocorticosteroids are a type of drug that can reduce SASP secretion and inflammation induced by senescent cells via reduction of transcriptional activity of NF-κB [4]. But several severe effects have emerged on the application of glucocorticosteroids as skin senolytics, for instance skin thinning and impaired wound healing [32]. For this reason, there is an urge to discover a safer and effective therapy from natural sources to combat senescence. Nowadays, the development of new anti-ageing drugs from natural sources has gained worldwide attention.

Polyphenols are naturally occurring compounds which can be found prevalently in fruits, vegetables, seeds, and spices, as well as in red wine, coffee and cocoa [33]. In addition, other natural sources like bee products are also active sources of polyphenols, besides proteins, vitamins, peptides, minerals, terpenes and fatty acids [34]. Supplementation of polyphenols have also showed positive effects as antioxidant and free radical scavenging activity, anti-tumour and anti-inflammatory properties and antithrombotic and anti-microbial activity [35]. Many research suggest that polyphenols may be able to stop or slow down ageing as well as ageing-related reduction in skin function and appearance through the vital cellular pathways for regulating cellular senescence and SASP [36].

## 3. Potential roles of bee product polyphenols in the prevention of senescence

**Fig. 2:**
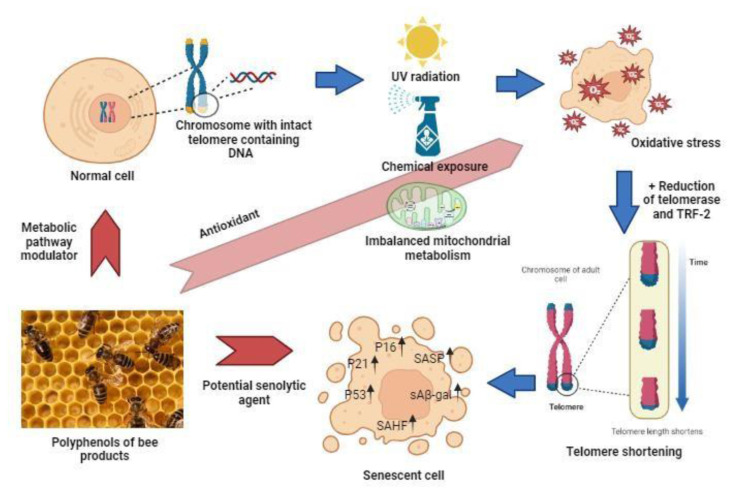
The potential role of polyphenols in bee products in prevention of cell senescence.

Many accumulated evidences supported that elimination of senescent cells and modulation of metabolic pathways in delaying senescence can reduce age-dependent deterioration in tissues and organs which leads to ageing [37]. There are number of potential senolytics, polyphenols, which are potent antioxidants, reported to perform both senescence delay and cell senescence elimination [38]. Polyphenols exert their actions as powerful metabolites in battling some of the ageing hallmarks, for instance mitochondrial dysfunction, telomere shortening, SIRT-1 deacetylase action, inflammation, autophagy, apoptosis and impaired protein homeostasis or proteostasis [39]. Fig. 2 gives an overview on the factors that lead to senescence and its prevention via polyphenols in bee products.

In particular, a report by Sharpless and Sherr [21] stated that cellular senescence or replicative senescence happens due to the shortening of telomeres after progressive proliferation in the absence of telomerase activity. Moreover, shortening of telomeres is a renowned theory that is often linked to ageing [39]. Oxidative stress and the free radicals’ attacks were assumed to be causative roles in telomere shortening through reduction of telomerase activity and telomere repeat binding factor 2 (TRF-2) level [40]. The regulation of telomere length to improve the health span during the ageing process is looked-for via healthy diet and physical activity [41,42]. Healthy diets such as consumption of antioxidant-rich foods can influence telomerase activity and lead to slow the telomere length shortening, a vital factor in postponing cellular senescence as well as ageing [43]. Besides, consumption of antioxidant supplements also might decelerate the shortening of telomere, which may lead to cellular senescence [44].

This review focusses on bee products as it is one of the natural nutraceuticals rich in antioxidants contributed by its polyphenolic compounds, primarily phenolic acids and flavonoids [34]. Nutraceuticals are ‘foods and food products’ that have therapeutic importance, principally in the prevention of age-related diseases [40]. In recent years, interest in discovering more about the advantages of bee products has been growing [45]. Bee products are products made by the bees including honey, propolis, royal jelly, bee pollen, bee bread, venom and wax [46]. Among that, honey is the most famous and widely appreciated bee product [47]. The different polyphenols present in bee products are shown in Table 1.

Honey is a type of natural food source consisting of carbohydrates, water and various bioactive compounds such as organic acids, amino acids, vitamins, minerals and wide range of polyphenols which are obtained from plants' nectar [48]. Honey is not only derived from the nectar of plants, but also from honey dew, which is essentially from plant secretions or excretions of sucking insects [49]. Honey as well as black cumin honey are rich in polyphenes such as benzoic acid, gallic acid, ellagic acid, *p*-coumaric acid, chrysin and pinocembrin [50]. Those polyphenes are grouped into two subclasses, phenolic acids and flavonoids. Polyphenolic compounds in honey varies across geographical area and their botanical sources [51]. The polyphenols in honey are essentially obtained from plants’ natural compounds [52]. These compounds mainly contribute to the antioxidant activities of honey [53], and because of that, honey is not only appreciated as a food or sweetener, but also has been applied as a medicine to cure many ailments since ancient times [54]. Furthermore, the colour intensity of honey is affected by its floral sources as well as its contents, pigments and chemical composition such as polyphenols [55]. The darker the colour of the honey, the higher the antioxidant activities [56]. Chestnut honey [57], oak honey [58], black cumin sativa honey [50], heather honey [57], and kelulut honey [59] are examples of dark-coloured honey with higher antioxidant capacities. Moreover, honey polyphenols not only serve as antioxidants but also contribute to a wide range of therapeutic effects such as antimicrobial, anti-inflammatory, anti-ageing and also other health benefits [60].

Propolis is a bee product composed of sticky plant substances or plant resins which is collected by the bees and processed in their hive [61]. The bees, particularly *Apis mellifera*, use propolis, a type of natural sealant, for their hive protection from extinction by the predators [62]. Propolis contains many compounds including polyphenols, flavonoids, aglycones, phenolics and ketone [63]. Among these compounds, flavonoids represent one-fifth of the polyphenols found in raw propolis [62], based on its total flavonoid contents. Flavonoids, the largest member of the polyphenol family, are agents with a variety of biological actions, including antibacterial, anti-inflammatory, and antioxidant effects [64]. Another study has shown that propolis ethanolic extract augmented the viability of senescent C2C12 cells, reduced the number of senescence associated β-galactosidase (SA-β-Gal)-positive cells and enhanced the differentiation of C2C12 cells [65] which were attributed to its coffee phenethyl ester (CAPE), and appears as a main active component in propolis and plays a role in activating the Nrf2 pathway, thereby balancing the redox state through the Nrf2/HO-1 pathway.

Another bee product, bee pollen also contains bioactive compounds including proteins, amino acids, lipids, carbohydrates, minerals, vitamins and polyphenols which can boost immunity, promote blood circulation, postpone ageing, improve health and stimulate both mental and physical activity [66]. Bee pollen is a mixture of pollen grains with the digestive enzymes of bees after they visit flowers and gather pollen grains on their feet to form pellets that build up in their hive [67]. Bee pollen is considered the “most natural perfect food” due to its great source of all essential amino acids required in honey bee and human nutrition [68]. It has been reported that the antioxidant activity of bee pollen comes from its high number of polyphenols [67].

Bee bread is a bee by-product created from pollen gathered by bees and combined with nectar and bee's salivary enzymes prior to lactic acid fermentation in beehives [69]. Bee bread is high in carbohydrate, protein, and fats, as well as minerals, vitamins, phenolic compounds, and essential amino acids [70].

Beeswax is a naturally occurring substance produced by wax-producing glands in worker bee's abdomen whose additive contents can vary depending on the bee's geographical location [71]. Beeswax contains a rich source of proteins, minerals and some major polyphenols, which confer strong antioxidant activity and low levels of toxicity [72].

Bee venom, or apitoxin, a secret weapon of honeybees, is a type of biotoxin produced in the venom gland of honeybees (Apidae family) under their abdominal cavity [73]. Bee venom is comprised of active peptides, with melittin as their major component besides apamin and adolapin, and enzymes (phospholipase A2, and hyaluronidase), as well as non-peptide components, such as histamine, dopamine, and norepinephrine [74]. It is reported to act as an anti-inflammatory, leishmanicidal, antimicrobial, antiviral, antiapoptotic, wound healer, antifibrinolytic and antielastolytic agent [75]. Besides peptides and enzymes, it also possesses other active substances like polyphenols, a powerful substance that can combat free radicals as reported earlier (Table 1).

Royal jelly, a gel-like substance secreted by the honeybees, is used to feed queen bees, owing health promoting effects including suppressing senescence [76]. Royal jelly treatment increased the proliferation of long-term culture of human primary epidermal keratinocytes (HPEKs), suggesting that royal jelly inhibits senescence. Royal jelly helps in suppressing senescence in HPEKs by regulating the expression levels of ΔNp63, p16, and p21 [76].

### 3.1. Polyphenols in bee products reduce oxidative stress

Oxidative stress is one of the mechanisms involved in cellular senescence. Oxidative stress is defined as an increase in the intracellular concentration of free radicals and ROS including superoxide anions, hydroxyl radicals and hydrogen peroxide generated during mitochondrial electron transport chain [88]. Although ROS possess some important protective roles such as defence body from opportunistic pathogens and aggravate the production of communication hormones between cells, overaccumulation of these radicals often causes ageing [89]. Oxidative stress portrays an imbalance between antioxidants and ROS [90]. Antioxidants are compounds that hinder cells from oxidative damages caused by ROS and free radicals through capture, stabilisation or deactivation free radicals before they reach cells [91]. In senescent cells, the production of ROS is significantly increased, hence the antioxidant mechanisms are vital to scavenge the intracellular levels of ROS and the occurrence of oxidative damage in senescent cells [13].

Honey, renowned as polyphenols reservoir owing to its antioxidant properties reduced oxidative stress caused by ROS formation. Honey has been incorporated into the substrate of poly-vinyl alcohol (PVA) nanofibre as scaffolds for wound healing and employed to reduce replicative senescence of umbilical cord-derived mesenchymal stem cells (UCDMSCs) [92]. The outcomes of the study showed that the UCDMSCs cultured on PVA honey substrates proliferated significantly and reduced its senescence parameter, both ROS and β-galactosidase (β-gal) markers, respectively, compared to cells cultured on pure PVA substrates. This indicates that PVA honey substrates exhibited antioxidant properties, which can relieve the replicative senescence of UCDMSCs. Another study showed that showed that culturing corneal progenitor cells with 0.0004–0.4% tualang honey lowered oxidative stress on the cells [93].

Propolis has also been reported to possess antioxidant activity attributed by their galangin and pinocembrin compounds [94]. It was widely acknowledged that phenolic components present in propolis prevent the oxidation of lipids, proteins, and nucleic acids by contributing hydrogen ions to free radicals [95]. In another study, it was reported that bee pollen and bee bread had a stronger antioxidant potential than honey and beeswax [73]. This is most likely caused by the abundance of bioactive substances found in bee pollen. Hence, these compounds can be considered as novel therapeutic agents with anti-ageing activity as it can reduce various diseases and increase life-span [96]. Despite this fact, there is still a challenge to find exact molecular mechanisms of capability of polyphenols in altering age-related diseases due to the complexity of diverse biochemical pathways all of which can contribute to ageing [97].

### 3.2. Polyphenols in bee products reduce telomere shortening

Telomere length is well-recognised as one of the top biomarkers for ageing [16]. Increase in telomere length helps to reduce the risk of functional senescence in cells that causes ageing. Numerous studies have shown that antioxidants may halt the accelerated telomere shortening driven by an increase in oxidative stress. Oxidative stress is thought to decrease the telomere length through decreasing the activity of telomerase, activity of TRF-2 levels and accumulation of oxidised guanines, 8-hydroxy-2-deoxyguanosine (8oxodG) [98]. The ability of polyphenols in enhancing telomerase activity and maintaining telomere length have made it a targeted antioxidant supplement by many researchers nowadays [99].

A study by Mohamad Nasir et al. [100] revealed that the consumption of bee products, including the period and frequency of consumption, influenced the telomere length of the beekeepers compared to non-beekeepers, with a mean increase in telomere length of 0.258 kbp and 2.66 kbp, respectively. The main functional component in royal jelly called major royal jelly proteins (MRJPs) has been reported to have anti-senescence activities on human cells when cultured with human embryonic lung fibroblasts (HFL-I) [101]. The cells in medium containing MRJPs had the highest proliferation activity, the lowest senescence and the longest telomere length.

Even though numerous studies have shown the relationship between telomere length and polyphenols, there is still lack of information regarding the mechanism how polyphenols in bee products primarily phenolic acids and flavonoids protect telomere length. Hence, further research on the mechanism of protection of honey polyphenols on telomere length and prevention of early cell senescence is necessary to extend longevity of life.

## 4. Conclusion and future perspectives

Among the stress related factors impacting cellular senescence, shortened telomeres appear to have gained acceptance as gold standard markers. Antioxidants, a type of chemical counteracting telomere shortening are seen as a potential source for slowing telomere shortening via removal of ROS and free radicals contributing to oxidative stress. Polyphenols, as reviewed in this article, are shown to possess unique anti-ageing intervention properties attributable to their antioxidant activities that delay senescence via modulation of metabolic pathways in cells apart from possible role in the removal of senescent cells. Bee products (honey, propolis, royal jelly, bee pollen, bee bread, venom and wax) are naturally polyphenol-rich food sources, have potent antioxidant properties attributable to its phenolic acids and flavonoids that can delay cell ageing and senescence. As such, phenolic acids and flavonoids sourced from bee products have the potential for use as novel anti-ageing drug from a natural source for combating ageing related maladies. However, an in depth understanding on how polyphenols influence telomere length is crucial as specific studies addressing this issue do not appear in the literature. Therefore, further insights into the role of polyphenols of bee products in prevention of telomere shortening are desired for a better understanding on the age-related diseases and wellness during longevity in humans.

## Figures and Tables

**Table 1:** Bee products containing polyphenols.

Type of bee product	Major polyphenols	Amount	Reference
Oak honey (Thrace, Turkey)	Protocatechuic acid	19.64 μg/g	[57]
	Rutin	26.37 μg/g	
	Caffeic acid	6.31 μg/g	
Black cumin honey (Burdur, Kayseri and Kilis, Turkey)	Ellagic acid	85.64 – 758.60 μg/g	[77]
	Pinosembrin	5.60 – 102.40 μg/g	
	Myricetin	1.08 – 26.70 μg/g	
	Quercetin	1.35 – 14.26 μg/g	
Stingless bee honey (Amazonas, Northern Brazil)	trans, trans-abscisic acid	0.57 – 1.60 mg/100ml	[78]
	cis, trans-abscisic acid	0.18 – 1.58 mg/100ml	
	Taxifolin	3.80 – 67.00 mg/100ml	
Propolis (Anatolian)	Pinocembrin	564 – 15374 μg/g	[62]
	CAPE	513 – 7310 μg/g	
	Chrysin	1152 – 15394 μg/g	
	Cinnamic acid	53 – 1922 μg/g	
Propolis (Poland, Romania, Turkey and Uruguay)	Chrysin	457.01 – 2817.43 μg/g	[79]
	Caffeic acid	89.61 – 778.37 μg/g	
	p-Coumaric acid	277.12 – 3547.56	
	CAPE	344.63 – 1118.62 μg/g	
Propolis (China and Brazil)	Pinobanksin 3-Oacetate	0.31 – 151.90 mg/g	[80]
	Pinocembrin	0.40 – 142.30 mg/g	
	Chrysin	0.19 – 59.10 mg/g	
	Galangin	0.26 – 49.00 mg/g	
	Pinobanksin	10.20 – 29.40 mg/g	
Bee pollen (Turkey)	t-cinnamic acid	560 – 6800 μg/100g	[67]
	Chrysin	177 – 1773 μg/100g	
	Pinocembrin	177 – 1773 μg/100g	
Bee pollen (Poland)	Rutin	0.20 – 5.34 mg/100g	[81]
	Salicyclic acid	0.12 – 2.57 mg/100g	
	Vanilin	0.07 – 0.23 mg/100g	
Bee pollen (Philippines)	Rutin	0.47 – 1.55 mg/g	[82]
	Quercetin-3-glucoside	1.43 – 3.39 mg/g	
	Quercetin	0.44 – 0.99 mg/g	
Stingless bee bread (Malaysia)	Caffeic acid, ferulic acid, kaempferol, apigenin and sorhamnetin	Caffeic acid, ferulic acid, kaempferol, apigenin and sorhamnetin	[83]
Bee bread (Romania)	Kaempferol	31.25 mg/l	[84]
	Myricetin	3.15 mg/l	
	Luteolin	1.17 mg/l	
Beeswax (Kujawy region, Poland)	Pinobanksin	85.70 μg/g	[85]
	Kaempferol	6.00 μg/g	
	Protocatechuic acid-O-hexoside	3.40 μg/g	
	Apigenin	3.00 μg/g	
	3,4-di-O-caffeoylquinic acid	1.90 μg/g	
Beeswax byproduct from beeswax recycling process (Italy)	Isorhamnetin derivatives	NS	[72]
	Kaempferol derivatives		
	Myricetin derivatives		
	Quercetin derivative		
	Tiliroside		
Bee venom (Aga, Egypt)	Apigenin	NS	[86]
	Genistein		
Royal jelly (Almería, Spain)	Ferulic acid	12946 – 18936 μg/kg	[87]
	Apigenin	408 – 930 μg/kg	
	Coumestrol	835 μg/kg	
	Naringenin	117 – 793 μg/kg	

NS: not stated
